# Biologic Propensities and Phytochemical Profile of *Vangueria madagascariensis* J. F. Gmelin (Rubiaceae): An Underutilized Native Medicinal Food Plant from Africa

**DOI:** 10.1155/2014/681073

**Published:** 2014-04-10

**Authors:** Nelvana Ramalingum, M. Fawzi Mahomoodally

**Affiliations:** Department of Health Sciences, Faculty of Science, University of Mauritius, 230 Réduit, Mauritius

## Abstract

*Vangueria madagascariensis* (VM), consumed for its sweet-sour fruits, is used as a biomedicine for the management of diabetes and bacterial infections in Africa. The study aims to assess the potential of VM on **α**-amylase, **α**-glucosidase, glucose movement, and antimicrobial activity. The antioxidant properties were determined by measuring the FRAP, iron chelating activity, and abilities to scavenge DPPH, HOCl, ^**∙**^OH, and NO radicals. Leaf decoction, leaf methanol, and unripe fruit methanol extracts were observed to significantly inhibit **α**-amylase. Active extracts against **α**-glucosidase were unripe fruit methanol, unripe fruit decoction, leaf decoction, and ripe fruit methanol, which were significantly lower than acarbose. Kinetic studies revealed a mixed noncompetitive type of inhibition. Leaf methanolic extract was active against *S. aureus* and *E. coli*. Total phenolic content showed a strong significant positive correlation (*r* = 0.88) with FRAP. Methanolic leaf extract showed a more efficient NO scavenging potential and was significantly lower than ascorbic acid. Concerning ^**∙**^OH-mediated DNA degradation, only the methanol extracts of leaf, unripe fruit, and ripe fruit had IC_50_ values which were significantly lower than **α**-tocopherol. Given the dearth of information on the biologic propensities of VM, this study has established valuable primary information which has opened new perspectives for further pharmacological research.

## 1. Introduction

*Vangueria madagascariensis *(VM) J. F. Gmelin (Rubiaceae), also commonly known as Vavangue, Voavanga, or Tamarind of the Indies, is a perennial plant which is native to tropical Africa and Madagascar [1]. Some species of genus* Vangueria* are widely studied* in vitro* and used in traditional medicine in various countries. For instance, in Tanzania, different parts of the species* Vangueria infausta* have traditionally been used for the treatment and/or management of malaria, wounds, menstrual, and uterine problems [2].

With respect to VM, available folk data suggest its use as an anthelmintic against roundworms, as antimicrobial, as astringent against cholagogue, and as expectorant, for the treatment of smallpox and sores, herpes labialis, and in the management of diabetes [3]. Preliminary phytochemical screening of the leaves and stems has shown the presence of alkaloids, terpenes, and cyonogenetic heterosides as well as phenols, tannins, and saponosides which may likely be responsible for its antimicrobial effects [1]. According to Musa et al. [4] roots of VM are macerated and administered orally for the treatment of diabetes mellitus. In Mauritius, an infusion of the leaves of VM, ingested once a week, has also been reported for the same purpose [1]. Moreover, a study carried out among Islanders of the Indian Ocean, which also included Mauritians, reported that leaf decoction is taken mainly to treat skin infections and abscesses [5].

There is currently a dearth of scientific validation of the purported traditional uses of VM as a biomedicine and previous evidence may still be considered as insufficient to support its folkloric use [5–7]. Additional research work is needed to probe into the antidiabetic, antimicrobial, and antioxidant properties of VM which may help validate its traditional claims and delineate further health benefits. Therefore, the main aim of this study was to investigate the antidiabetic, antimicrobial, and antioxidant properties of the leaves, ripe and unripe fruits, and the seeds of VM. To the best of our knowledge this is the first study to report the biological activity of VM* in vitro*. Given the dearth of updated information on the biological properties of VM, this work can provide an opportunity to establish valuable primary information on the bioactivity of VM and hence open new perspectives for further pharmacological research.

## 2. Materials and Methods 

### 2.1. Preparation of Plant Materials

Fresh leaves and both unripe and ripe fruits of VM were collected from Black River, Mauritius. They were authenticated at the National Herbarium of the Mauritius Sugar Industry Research Institute, Réduit. Fruits were cut into small pieces and seeds were removed on the day of collection. The mesocarp and epicarp pieces of fruits were lyophilized overnight whilst the seeds were crushed to remove the endocarp. The endocarp was discarded and the seeds along with the leaves were air dried under shade for 5–7 days till constant mass was obtained. The dried leaves, seeds, and pieces of fruits were homogenized in an electrical food grinder to a fine powder and were stored in air-tight containers.

### 2.2. Extraction Process

Methanol (Sigma-Aldrich, St. Louis, USA) and decoction extracts were used in the current study. It was important to assess the therapeutic properties of the crude extract in order to validate the medicinal uses of the different parts of the plants, as this is the way in which the local population uses them. All extracts were concentrated* in vacuo* until a constant weight was obtained and the percentage (%) yield was calculated [8]. The gummy material was collected and stored in tightly closed bottles in the dark at 4°C for biological assays.

### 2.3. *In Vitro* *α*-Amylase Assay

The activity of *α*-amylase was carried out according to the starch-iodine colour changes with minor modifications [7]. Briefly, 0.1 mL of *α*-amylase solution (15 *μ*g/mL in 0.1 M acetate buffer, pH 7.2 containing 0.0032 M sodium chloride) was added to a mixture of 3 mL of 1% soluble starch solution (1 g soluble potato starch, suspended in 10 mL water was boiled for exactly 2 min. After cooling, water was added to a final volume of 100 mL. The solution was kept in the refrigerator and was used within 2-3 days) and 2 mL acetate buffer (0.1 M, pH 7.2) preequilibrated at 30°C in a water bath. Substrate and *α*-amylase blank determinations were undertaken under the same conditions. At zero time and at the end of the incubation period, 0.1 mL of reaction mixture was withdrawn from each tube after mixing and discharged into 10 mL of an iodine solution (0.245 g iodine and 4.0 g Potassium Iodide in 1 liter). After mixing, the absorbency of the starch-iodine mixture was measured spectrophotometrically at 565 nm. The absorbency of the starch blank was subtracted from the sample reading. One unit of amylase activity was arbitrarily defined as [*A*_0_ − *A*_*t*_/*A*_0_] × 100, where *A*_0_ and *A*_*t*_ were absorbances of the iodine complex of the starch digest at zero time and after 60 min of hydrolysis. Specific activity of amylase was defined as units/mg protein/60 min. Extract (0.10 mL) was incubated with 0.1 mL of the enzyme and substrate solution for 15 min at 30°C. The assay was conducted as described above; one unit of amylase inhibitor was defined as that which reduced the activity of the enzyme by one unit. Assays were replicated three times and the mean values were used. The percentage *α*-amylase inhibition was calculated according to the formula [9]:

(1) %  inhibition ={absorbance(control)−absorbance(sample)}absorbance(control)×100%.

### 2.4. *In Vitro* *α*-Glucosidase Assay

The *α*-glucosidase inhibitory activity was determined as described previously [10, 11]. The inhibition was measured spectrophotometrically (405 nm) in the presence of the extracts or positive control (20 *μ*L at varying concentrations) at pH 6.9. In a 96-microtitre plate, a reaction mixture containing extracts, 20 *μ*L of 1 mM* p*-nitrophenyl *α*-D-glucopyranoside as a substrate and 1 unit/mL glucosidase enzyme, in 50 *μ*L of 0.1 M sodium phosphate buffer was preincubated for 30 min at 37°C. After incubation the reaction was stopped by adding 50 *μ*L of sodium carbonate (0.1 M). Acarbose (400 *μ*g/mL) was used as a positive control. The IC_50_ value was defined as the concentration of *α*-glucosidase inhibitor to inhibit 50% of its activity under the assay conditions.

### 2.5. Kinetic Studies

Kinetic studies were carried out according to Kotowaroo et al. [7] with minor modifications. A concentration of 0.10 g/mL of the extracts was used and a calibration curve was constructed using a modified glucose-based colorimetric assay [7]. A 1% dinitrosalicyclic solution (DNS) was prepared by mixing 10 g of dinitrosalicyclic, 0.5 g sodium disulphite and 10 g NaOH in 1 L distilled water. 3 mL of this solution was then added to glucose solution at different concentration (10, 5, 2.5, 1.25 and 0.625 g/L). The test tubes, covered with paraffin film were heated at 90°C for 5–15 min until a red brown coloration developed. 1 mL of 40% Rochelle salt solution was then added. The test tubes were cooled under tap water and the absorbance was measured at 575 nm. A double reciprocal plot (1/*V* versus 1/[*S*]) where *V* is reaction velocity and [*S*] is substrate concentration was plotted.The kinetic constants (*K*_*m*_ and *V*_max⁡_) were calculated [12], where *K*_*m*_ is the Michaelis-Menten constant, *V*_max⁡_ is the maximal velocity, [*S*] is the substrate concentration, and *V* is the rate of reaction.

Evaluation of the kinetics parameters of *α*-glucosidase inhibition by the plant extracts was conducted as described previously [10, 13] with minor modifications. Enzyme activity was measured with increasing concentrations of* p*-nitrophenyl *α*-D-glucopyranoside (PNPG) (0.0625, 0.125, 0.25, 0.5, and 1 mM) as substrate in the absence or presence of the plant extracts at a single concentration. Plant extract was incubated with 10 *μ*L**α**-glucosidase solution (1 U/mL), 50 *μ*L sodium phosphate buffer (0.1 M, pH 6.9), and 20 *μ*L graded concentrations of PNPG for 30 min at 37°C. The reaction was terminated by adding 50 *μ*L sodium carbonate (0.1 M). The velocity of the reaction was defined as the rate of formation of the product,* p*-nitrophenol, which was determined using a calibration curve constructed by measuring the absorbance of varying concentration of* p*-nitrophenol.

### 2.6. Glucose Movement* In Vitro*

A simple model system was used to evaluate effects of VM extracts on glucose movement* in vitro *based on a modified method [14]. This method involved the use of a dialysis tube (10 cm × 15 cm) into which 2 mL of a solution of glucose (22 mM) and NaCl (0.15 M) and 1 mL of plant extract (20 mg/mL) were introduced and sealed. The tube was placed in a conical flask containing 40 mL of 0.15 M NaCl solution with 10 mL distilled water. The conical flask was then placed in an orbital shaking incubator at 37°C on 100 rpm. The appearance of glucose in the external solution was measured at set time intervals. The effects of plant extract on glucose diffusion were compared to control tests conducted in the absence of plant extracts. All tests were carried out in triplicate.

### 2.7. Antimicrobial Screening

The procedures used for the antimicrobial screening in the present study are as described previously [15, 16]. The disc diffusion method was used as a preliminary test to find out if plant extracts were active. Clear inhibition zones around discs indicated the presence of antimicrobial activity. Inhibition zones less than 7 mm were not evaluated. If extracts show antimicrobial activity by disc diffusion, MIC (minimum inhibitory concentration) was then determined. MIC which is the least concentration of antimicrobial agent that will inhibit visible growth of an organism after an overnight incubation was determined using microtitre dilution broth method in 96-well microplates [17]. Streptomycin sulphate and gentamicin sulphate were used as positive control for testing against* S. aureus* and* E. coli*, respectively.

### 2.8. Antioxidant Activities

*DPPH Free Radical Scavenging Assay.* Assay was carried as described previously [18]. Stock solutions of crude extracts and the positive control, ascorbic acid (400 *μ*g/mL), were prepared in methanol at appropriate concentrations and added to DPPH (200 *μ*L at 100 *μ*M prepared in methanol) in a 96-microtitre plate. The plate was then incubated for 30 min at 37°C. Absorbance of each solution was measured at 517 nm. The extracts and standard were analysed in triplicate at different concentrations and the IC_50_ values were determined as follows [19]:

(2) %  inhibition =absorbance  blank  sample−absorbance  extractabsorbance  blank  sample×100.

*Ferric Reducing Antioxidant Power (FRAP) Assay.* The FRAP assay was adapted from the method of Benzie and Strain [20]. The stock solutions included acetate buffer (300 mM, pH 3.6), TPTZ (10 mM) solution in HCl (40 mM), and FeCl_3_·6H_2_O solution (20 mM). The fresh working solution was prepared by mixing 25 mL acetate buffer, 2.5 mL TPTZ solution, and 2.5 mL FeCl_3_·6H_2_O solution and then equilibrating at 37°C for 15 min before using. Plant extracts (0.15 mL) at known concentrations were allowed to react with FRAP solution (2.85 mL) for 30 min in the dark. Analysis of extracts and positive control trolox (200 mM) were done in triplicate. Readings of the Persian blue complex were then taken at 593 nm. Results were expressed in mM trolox equivalent (TE)/g fresh mass using the following equation based on the calibration curve: *y* = 0.0016*x*, *R* ^2^ = 0.8336.

*Hypochlorus Acid (HOCl) Scavenging Assay.* HOCl was measured by the chlorination of taurine [21]. Sample cuvettes contained HOCl (100 *μ*L; 600 *μ*mol/L), taurine (100 *μ*L; 150 mmol/L), and 100 *μ*L of plant extracts at various concentrations in a total volume of 1 mL of PBS at a pH of 7.4. The reaction mixtures were thoroughly mixed and then allowed to stand for 10 min at room temperature. After incubation, potassium iodide solution (100 *μ*L; 20 mmol/L) was added and absorption was measured against reference blank cuvette (100 *μ*L PBS instead of extract; absorbance corresponding to 100% HOCl) at 350 nm. The absorbance of the reaction mixture was read both before and after the addition of potassium iodide. The results were expressed as the percentage HOCl inhibitions for each extract and the positive control; ascorbic acid (400 *μ*g/mL). The IC_50_ was calculated.

*Hydroxyl Radical* (^∙^OH)* Scavenging/Deoxyribose Assay.* ^∙^OH scavenging activity was assessed by determining its ability to oxidise deoxyribose [22]. The reaction mixture consisted of 100 *μ*L of hydrogen peroxide (15 *μ*mol/L), 100 *μ*L iron chloride (3 mmol/L), 100 *μ*L EDTA (3 mmol/L), 100 *μ*L ascorbic acid (3 mmol/L), and 100 *μ*L extracts at various concentrations. 2-Deoxy-ribose (100 *μ*L) was then added followed by PBS (pH 7.4) in a total volume of 1 mL. After 30 min of incubation at 37°C, 60% trichloroacetic acid (1 mL) and thiobarbituric acid (0.5 mL; 1 g in 100 mL of 0.05 mol/L sodium hydroxide) were added to the reaction mixture. The reaction mixture was boiled for 20 min to observe the development of light pink chromogen. After boiling the absorbance was measured at 532 nm and the ^∙^OH scavenging activity of the extract was reported as the percentage of inhibition of deoxyribose degradation against *α*-tocopherol (400 *μ*g/mL) as positive standard. The IC_50_ was calculated.

*Nitric Oxide Radical (NO) Scavenging Assay.* At physiological pH, nitric oxide generated from aqueous sodium nitroprusside solution (SNP) interacts with oxygen to produce nitrite ions, which may be quantified by Griess Illosvay reaction [23]. The reaction mixture (3 mL) contained SNP (2 mL 10 mM), PBS (0.5 mL), and extract and standard solution at various concentrations (0.5 mL). The mixture was incubated for 25°C for 150 min after which 0.5 mL was transferred and mixed with 1 mL sulphanilic acid reagent (0.33% in 20% glacial acetic acid) and allowed to stand for 5 min for complete diazotization. Naphthyl Ethylenediamine dihydrochloride (1 mL; 0.1%* w/v*) was added, mixed, and allowed to stand for a further 30 min. The pink-coloured chromophore was measured spectrophotometrically at 540 nm against a blank sample. All tests were performed in triplicates and ascorbic acid (400 *μ*g/mL) was used as positive standard. The IC_50_ was calculated.

*Iron Chelating Activity.* The ability of the various extracts to chelate Fe (II) was investigated using a modified method [24]. The principle is based on the formation of a purple coloured complex, which is inhibited in the presence of chelating agents. The reaction mixture contained 200 *μ*L of the plant extract of varied concentration and 50 *μ*L of ferric chloride/FeCl_2_·4H_2_O (2.5 mM), which was made up to 1 mL by the addition of deionised water and was incubated for 5 min at room temperature. Ferrozine (50 *μ*L of 2.5 mM) was then added, and the absorbance was read at 562 nm. EDTA (400 *μ*g/mL) was used as positive control. Percentage chelating activity was calculated using the formula shown below. The IC_50_ was calculated

(3) %  chelating  activity =absorbance  blank−absorbance  sampleabsorbance  blank×100.

### 2.9. Quantitative Phytochemical Determination

*Total Phenol Content.* The total phenolic content was determined according to the Folin and Ciocalteu's method [25] with slight modifications. The extracts (0.5 mL; stock solution 1 mg/mL) were mixed with ten-fold diluted Folin-Ciocalteau's reagent (2.5 mL) into test tubes and aqueous sodium carbonate (2 mL, 7.5%) was added. The mixture was thoroughly mixed and allowed to stand for 30 min at room temperature. The resulting blue coloration was measured at 760 nm. All determinations were performed and results expressed in mg gallic acid equivalent (GAE)/g fresh weight using the calibration graph: *y* = 0.0036*x*, *R* ^2^ = 0.9341.

*Total Flavonoid Content.* Total flavonoid content was determined using a method of Amaeze et al. [25]. 2 mL plant extract was added to 2 mL of 2% AlCl_3_ solution which was prepared in ethanol. The absorbance was measured at 420 nm after being allowed to stand 1 hr at room temperature. All determinations were performed in triplicates and total flavonoid content was calculated as rutin equivalent (RE) in mg/g fresh weight based on the calibration curve: *y* = 0.0088*x*, *R* ^2^ = 0.9003.

*Total Proanthocyanidin Content.* Determination of proanthocyanidin content was carried out as reported previously [25]. The extract (0.5 mL) at various concentrations was mixed with 1.5 mL of 4% vanillin-methanol solution and 0.75 mL concentrated hydrochloric acid. The mixture was allowed to stand for 15 min after which the absorbance was measured at 500 nm. Total proanthocyanidin contents were expressed as catechin equivalents (CE) (stock solution 400 *μ*g/mL) using the following equation based on the calibration curve: *y* = 0.0015*x*, *R* ^2^ = 0.9025.

### 2.10. Qualitative Phytochemical Screening

The preliminary screening for different phytochemicals was based on the intensity of colour development formation of any precipitate on addition of specific reagents screening using modified standard protocols [26]. Results were reported as low amount (+), moderate amount (+ +), and high amount (+ + +) depending on intensity of colour formation [27].

### 2.11. Statistical Analysis

All data were expressed as means ± SD for three experiments. Statistical analyses were performed using SPSS version 16.0. Normality test was performed before using parametric tests (ANOVA or Pearson correlation). Normality tests were based on Shapiro Wilk's test where a *P* value >0.05 translates into normal data. ANOVA with Tukey multiple comparisons were carried out to test for any significant differences between the means. Correlations were obtained by Pearson correlation coefficient. The significance level was at 0.05 (*P* < 0.05) [28].

## 3. Results

Extraction of 50.0 g of powered plant materials with methanol resulted in slightly higher yield compared to decoction. The percentage yields of decoction were leaf extract (19.4%), unripe fruit extract (26.8%), ripe fruit extract (29.4%), and seed extract (8.2%). In contrast, the percentage yields for the methanolic extracts were leaf (20.0%), unripe fruit (24.2%), ripe fruit (29.8%), and seeds (19.8%).

### 3.1. Inhibitory Activity on Key Carbohydrate Hydrolyzing Enzymes

Leaf decoction (IC_50_ = 1.12 ± 0.17 mg/mL), leaf methanol (IC_50_ = 1.70 ± 0.10 mg/mL), and unripe fruit methanol (IC_50_ = 1.23 ± 0.24 mg/mL) extracts displayed the highest inhibitory activity (Table 1). However, the values were significantly higher than the positive control acarbose (IC_50_ = 0.11 ± 0.03 mg/mL). The weakest activity was observed in the ripe fruit (IC_50_ = 29.62 ± 13.73 mg/mL) and seed (IC_50_ = 6.81 ± 2.95 mg/mL) decoction extracts. Additionally, Table 1 also summarizes the effects of the different extracts of VM on *α*-glucosidase activity. The most active extracts (1 mg/mL) were unripe fruit methanolic extract (IC_50_ = 0.36 ± 0.07 mg/mL), unripe fruit decoction (IC_50_ = 0.50 ± 6.0 mg/mL), leaf decoction (IC_50_ = 0.61 ± 0.21 mg/mL), and ripe fruit methanol (IC_50_ = 3.28 ± 0.45 mg/mL), where values were significantly lower than acarbose (IC_50_ = 5.03 ± 0.14 mg/mL).

### 3.2. Kinetic Studies

**Figure 1:**
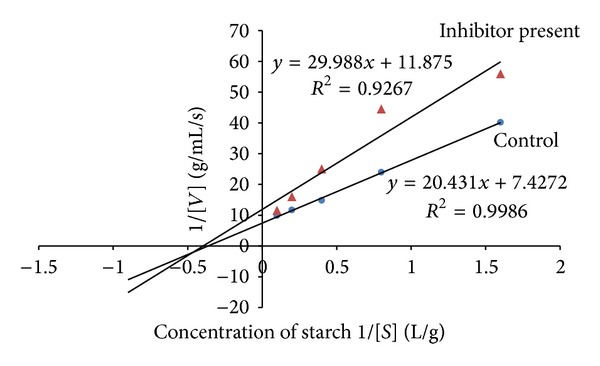
The Lineweaver-Burk plots for amylase in the presence or absence of leaf decoction extract (10 mg/mL). Each point represents values in the presence of the inhibitor: red triangle or control blue circle.

**Figure 2:**
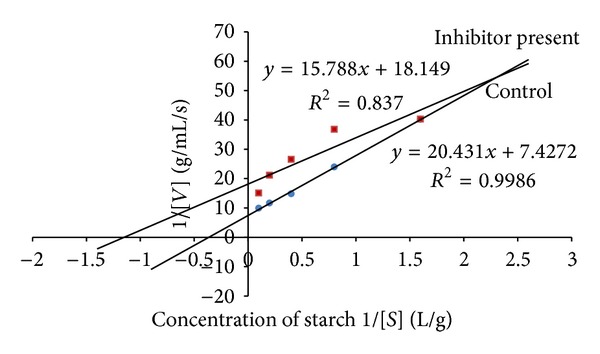
The Lineweaver-Burk plots for amylase in the presence or absence of leaf methanolic extract (10 mg/mL). Each point represents values in the presence of the inhibitor: red square or control blue circle.

**Figure 3:**
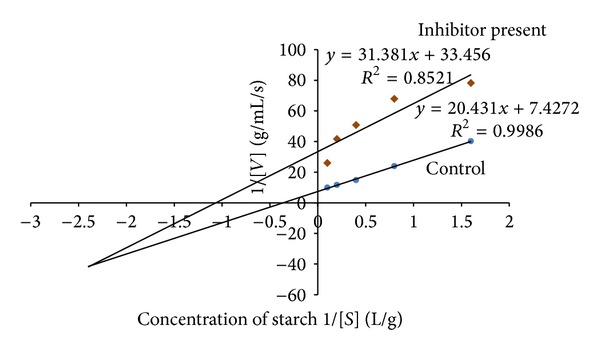
The Lineweaver-Burk plots for amylase in the presence or absence of unripe methanolic extract (10 mg/mL). Each point represents values in the presence of the inhibitor: brown diamond or control blue circle.

The leaf decoction, leaf methanolic, and unripe fruit methanolic extracts were assessed through kinetic studies to determine the type of enzyme-inhibition. The Lineweaver-Burk plots (Figures 1, 2, and 3) were generated using the calibration curve of glucose (*y* = 0.1254*x* + 0.4313). The double reciprocal Lineweaver-Burk plots showed a decrease in both *V*_max⁡_ (leaf decoction from 0.13 to 0.084 g/mL/s; leaf methanol extract from 0.13 to 0.055 g/mL/s; unripe methanol extract from 0.13 to 0.030 g/mL/s) and *K*_*m*_ (leaf decoction from 2.75 to 2.53 g/L; leaf methanol extract from 2.75 to 0.87 g/mL/s; unripe methanol extract from 2.75 to 0.94 g/mL/s) values when the inhibitor was added to the reaction mixture, confirming a mixed noncompetitive type of inhibition.

**Figure 4:**
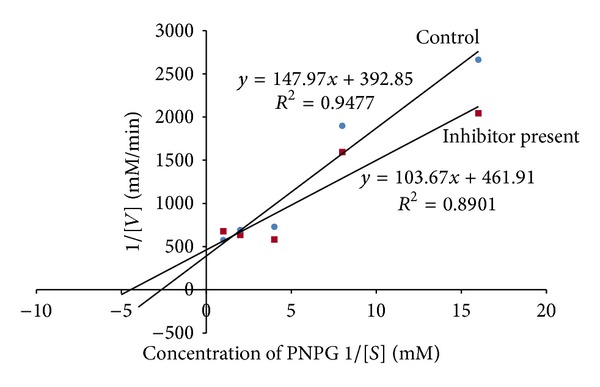
The Lineweaver-Burk plots for glucosidase in the presence or absence of leaf decoction extract (1 mg/mL). Each point represents values in the presence of the inhibitor: red square or control blue circle.

**Figure 5:**
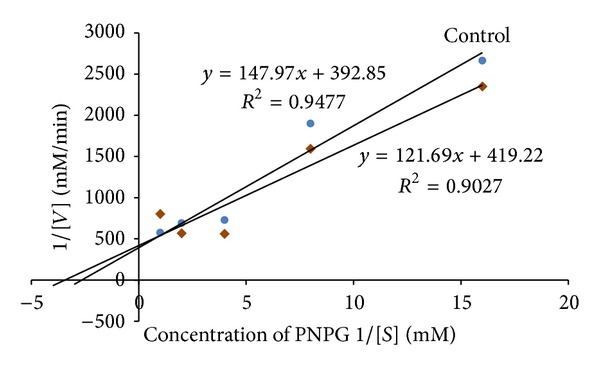
The Lineweaver-Burk plots for glucosidase in the presence or absence of unripe fruit decoction extract (1 mg/mL). Each point represents values in the presence of the inhibitor: brown diamond or control blue circle.

**Figure 6:**
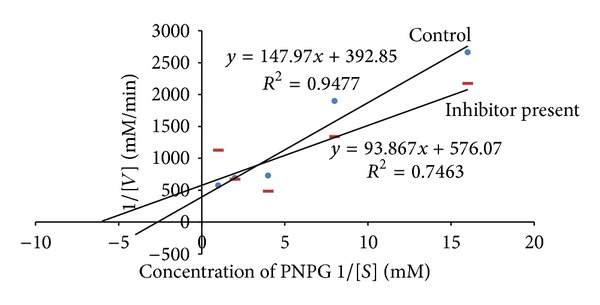
The Lineweaver-Burk plots for glucosidase in the presence or absence of unripe fruit methanol extract (1 mg/mL). Each point represents values in the presence of the inhibitor: red dash or control blue circle.

**Figure 7:**
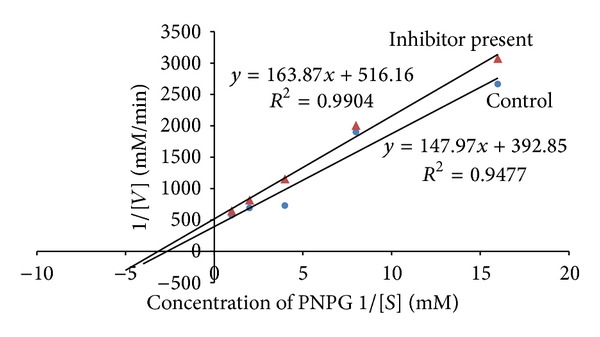
The Lineweaver-Burk plots for glucosidase in the presence or absence of ripe fruit methanol extract (1 mg/mL). Each point represents values in the presence of the inhibitor: red triangle or control blue circle.

Figures 4–7 show Lineweaver-Burk plots obtained by evaluating the leaf decoction, unripe fruit decoction, unripe fruit, and ripe fruit methanolic extracts through kinetic studies against *α*-glucosidase. The double reciprocal Lineweaver-Burk plots showed a decrease in both *V*_max⁡_ and *K*_*m*_ values when the extract was added. Such results suggest a mixed noncompetitive type of inhibition. From Figure 4, *V*_max⁡_ was observed to decrease from 0.0025 to 0.0021 mM/min while *K*_*m*_ decreased from 0.38 to 0.22 mM in the presence of leaf decoction extract. Also, *V*_max⁡_ decreased from 0.0025 to 0.0024 Mm/min while *K*_*m*_ decreased from 0.38 to 0.29 mM in the presence of unripe fruit decoction extract (Figure 5). With regard to unripe methanol extract, *V*_max⁡_ was found to decrease from 0.0025 to 0.0017 mM/min while *K*_*m*_ decreased from 0.38 to 0.16 mM (Figure 6). In the presence of ripe methanol extract, *V*_max⁡_ value was found to decrease from 0.0025 to 0.0019 Mm/min while *K*_*m*_ decreased from 0.38 to 0.32 mM (Figure 7).

### 3.3. Correlation of Carbohydrate Enzymes Inhibitory Effects with Phytochemical Constituents

The relationship between key carbohydrate enzymes inhibitory effects of extracts with total phenolic, flavonoid, and proanthocyanidin contents was investigated using Pearson correlation. As displayed in Table 2, there was no significant correlation (*P* > 0.05) with total phenolic, flavonoid, or proanthocyanidin content. However, the percentage shared variance for *α*-amylase was as follows: 4.0% for total phenolic content, 98.0% for total flavonoid content, and 31.4% for total proanthocyanidins. For *α*-glucosidase, the percentage shared variance was 96.0% for total phenolic content, 32.5% for flavonoid, and 98.0% for proanthocyanidin content.

### 3.4. Effect of VM Extracts on Glucose Movement* In Vitro*

Results in Table 3 revealed that most of the extracts did not significantly retard glucose movement across the dialysis tube. However, leaf decoction extract was the most active inhibitor of glucose movement in the model system where glucose diffusion was significantly decreased after 2 hr incubation period compared to control and external glucose concentration was 3.76 ± 0.080 mmol/L after 3 hr. In contrast, leaf methanol extract could decrease glucose movement earlier at a 1 h period of incubation but the overall decrease by a 3 hr period was significantly less (5.04 ± 0.046 mmol/L) compared to the decoction extract. Unripe fruit decoction extract (4.54 ± 0.046 mmol/L) could also significantly decrease glucose movement after 3 hr compared to control (7.16 ± 0.16 mmol/L) as well as leaf methanol extract (5.04 ± 0.046 mmol/L). However, though the movement of glucose was slow at the beginning and increased with time, such movement was not time dependent for the different extracts since overall no significant differences were noted in glucose concentrations between incubation times.

Results obtained for the antimicrobial tests performed on both the decoction and methanolic extracts of VM are presented in Table 4. It was found that the extracts showed a narrow spectrum of activity, being active only to the Gram positive* S. aureus *and to the Gram negative* E. coli. *Highest inhibitory activity was noted for* E. coli* using unripe fruit decoction extract (12.67 ± 0.58 mm), whereas for* S. aureus*, leaf methanol extract produced highest inhibition (11.67 ± 1.53 mm). However, no comparable zones of inhibition to respective standard antibiotic were obtained since mean inhibitory zones of inhibition for all active extracts were significantly lower (*P* < 0.05) than the mean standard.

### 3.5. Antimicrobial Screening by Disc Diffusion

The extracts showing antibacterial activities by disc diffusion method were tested by broth dilution assay to determine the MICs (Table 5). The lowest MIC value (6.25 mg/mL) was recorded for the methanolic leaf extract against* S. aureus *which can be considered as poor activity compared to the standard antibiotic.

### 3.6. Antioxidant Activities of Plant Extracts

#### 3.6.1. DPPH Radical Scavenging Assay

DPPH radical scavenging activity of the methanol extracts was higher compared to decoction extracts as the overall concentration of extracts needed to scavenge 50% DPPH radical was lower (Table 6). One way ANOVA analysis revealed that a significant difference exists between the different extracts (*F* = 349.97; *P* < 0.05). Post hoc comparison using Tukey HSD shows that the activity of all decoctions extracts was significantly different from their respective methanol extracts (*P* < 0.05). Moreover, only the activity of methanol extracts of leaf and unripe and ripe fruit was comparable to the positive control ascorbic acid (IC_50_ = 0.001 ± 0.0006 *μ*g/mL).

#### 3.6.2. Ferric Reducing Antioxidant Power of Extracts

Table 7 shows that there is a significant difference (*F* = 186.81; *P* < 0.05) between the antioxidant capacity of the extracts as assessed by FRAP. The different extracts were found to be active in the reduction of Fe^4+^ to Fe^2+^, indicating their antioxidant activity as reducing agents. The order of activity is as follows: leaf_methanol_ →  unripe  fruit_methanol_ → ripe  fruit_methanol_ → seed_methanol_ → leaf_decoction_ → unripe  fruit_decoction_ → ripe  fruit_decoction_ → seed_decoction_. Furthermore, a significant difference (*P* < 0.05) was noted between the unripe and ripe fruit decoction extracts and between the unripe fruit and ripe fruit methanolic extracts.

#### 3.6.3. Correlation between Antioxidant Activity and Phytochemical Content

The relationship between antioxidant activity with total phenolic, flavonoid, and proanthocyanidin contents was investigated using Pearson correlation. As displayed in Table 8, there was a strong, significant, negative correlation between total phenolic content and DPPH radical scavenging activity (*r* = −0.77, *P* < 0.05), implying that higher total phenolic content resulted in a lower concentration of extracts needed to achieve 50% scavenging activity. On the other hand, no statistically significant correlations were found between DPPH activity and total flavonoids and proanthocyanidins contents. The percentage of shared variance was only 8.0% and 16.3% between DPPH and total flavonoid and between DPPH and total proanthocyanidins, respectively. Also, with respect to FRAP assay, a strong significant positive relationship was found only with total phenolic content (*r* = 0.88, *P* < 0.05). The percentage of shared variance was 23.8% between FRAP and total flavonoid, whereas between FRAP and total proanthocyanidins it was 29.4%. Correlation between the DPPH method and FRAP method, however, revealed a strong significant negative relationship (*r* = −0.94, *P* < 0.05), meaning that these methods were reliable in assessing the antioxidant power of the extracts.

#### 3.6.4. HOCl Scavenging Activity

**Figure 8:**
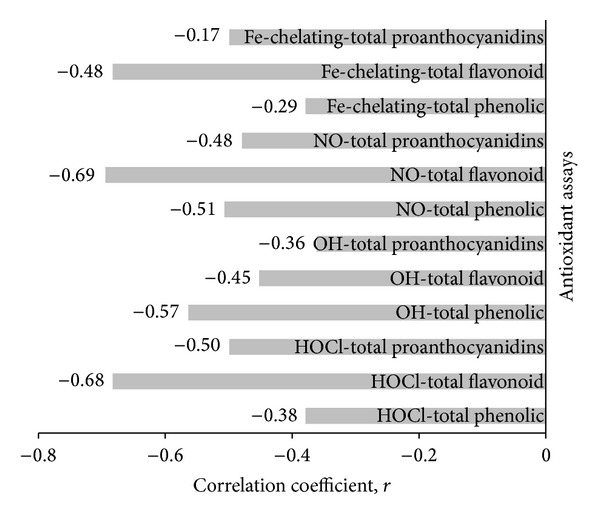
Correlation coefficients between iron-chelating activity, ^∙^OH, NO, and HOCl assays, with total phenolic, flavonoid, and proanthocyanidins contents.

Table 9 shows the HOCl scavenging activity of the different extracts of VM. Significant differences were only obtained between methanol and decoction extracts of ripe fruit and seed. Methanol unripe fruit extract had the highest scavenging action in view of its low IC_50_ value (IC_50_ = 222.99 ± 3.15 *μ*g/mL). However, none of the extracts had IC_50_ value that was greater than the control ascorbic acid (IC_50_ = 46.00 ± 2.35 *μ*g/mL). Also, seed decoction extract had the lowest value since its IC_50_ value (IC_50_ = 6656.35 ± 390.40 *μ*g/mL) was significantly higher than ascorbic acid. Correlation of this assay results with phytochemical content of the extracts (Figure 8) showed the strongest association with total flavonoid content (*r* = −0.68; 46.6% shared variance).

#### 3.6.5. ^∙^OH Scavenging Activity

All decoction extracts were significantly different (*P* < 0.05) from their respective methanol extracts in inhibiting ^∙^OH-mediated deoxyribose degradation (Table 9). The ripe fruit decoction extract was also significantly less active (*P* < 0.05) in scavenging ^∙^OH compared to the unripe decoction extract since a higher IC_50_ value was obtained (IC_50_ = 260.96 ± 4.29 *μ*g/mL). Moreover, compared to the positive control *α*-tocopherol, only the methanol extracts of leaf (IC_50_ = 0.09 ± 0.04 *μ*g/mL), unripe (IC_50_ = 0.29 ± 0.08 *μ*g/mL), and ripe (IC_50_ = 0.26 ± 0.02 *μ*g/mL) fruits which had IC_50_ values which were smaller than that of *α*-tocopherol (IC_50_ = 0.50 ± 0.11 *μ*g/mL). This suggests that they exhibited more efficient inhibitory activity than *α*-tocopherol. From Figure 8, correlation of ^∙^OH scavenging activity with quantitative evaluation of phytochemical content revealed a moderate negative relationship (*r* = −0.57) with total phenolics which translates into 32.5% of shared variance with total phenolic content.

#### 3.6.6. NO Scavenging Activity

As per Table 9, significant differences (*P* < 0.05) were only found between methanol and decoction extracts of ripe fruit and seed. Methanol leaf extract had an IC_50_ value (IC_50_ = 43.22 ± 0.59 *μ*g/mL) significantly lower than the control ascorbic acid (IC_50_ = 546.54 ± 9.79 *μ*g/mL) demonstrating a more efficient scavenging potential than the latter. The NO scavenging potential of decoction extracts of ripe fruit and unripe fruit was also significantly different. Strong negative correlation (*r* = −0.69; 47.6% shared variance) was also obtained with total flavonoid content (Figure 8).

#### 3.6.7. Iron Chelating Activity

From Table 9 it can also be observed that all the extracts had considerable iron chelating activity as demonstrated by their IC_50_ values (expressed in mg/mL) which are comparable to the positive control EDTA (IC_50_ = 0.001 ± 0.0003 *μ*g/mL). The strongest correlation (*r* = −0.48) was with total flavonoid content which resulted in 23.0% of shared variance.

### 3.7. Quantitative Phytochemical Analysis

Table 10 shows the overall mean concentration of total phenol, flavonoids, and proanthocyanidins. According to Tawaha et al. [29] plant species having GAE greater than 20 mg/mL dry weight were considered as having high phenolic content. It was noted that all samples were high in total phenol content, with VM leaf methanol extract having the greatest concentration. Post hoc analysis demonstrated that all decoction extracts were significantly different from their respective methanolic extracts. With regard to total flavonoid content, the concentration was found to vary between 6.72 ± 0.04 mg RE/g fresh weight and 8.90 ± 0.35 mg RE/g fresh weight for the decoction extracts and between 7.13 ± 0.13 mg RE/g fresh weight and 9.00 ± 0.05 mg RE/g fresh weight for methanol extracts. Significant difference (*P* < 0.05) was noted between flavonoid content of decoction and methanol extracts of unripe fruit. Also, the methanol unripe fruit sample shows significant difference (*P* < 0.05) compared to the methanol ripe fruit sample.

For total proanthocyanidins (*F* = 563.37; *P* < 0.05), comparison of mean within extraction solvent revealed that ripe fruit methanol extract significantly differed from the unripe fruit methanol extract (*P* < 0.05) as well as their respective decoction extracts. Proanthocyanidins content of all decoction extracts was also found to be significantly different from their respective methanol extracts.

### 3.8. Qualitative Phytochemical Screening

Table 11 shows the qualitative phytochemical screening of the different plant parts. Results were expressed as low amount (+), moderate amount (+ +), high amount (+ + +), or absence (−) to report the presence or absence of bioactive components. Phenolic compounds, flavonoids, and anthocyanins were present in all extracts.

## 4. Discussion

In the present series of* in vitro* experiments, the antidiabetic properties of the decoction and methanol extracts of different parts of VM were assessed in terms of their propensity to inhibit key intestinal carbohydrate digesting enzymes, namely, *α*-amylase and *α*-glucosidase. Consequently, the mode of enzyme inhibition for the most active extracts was determined using the Michaelis-Menten constant and maximal velocity in the presence and absence of the plant extracts. Findings in this study tend to demonstrate that only the leaf decoction, leaf methanol, and unripe methanol extracts exhibited significant inhibitory effects on *α*-amylase and *α*-glucosidase activity comparable to acarbose. Acarbose, being structurally analogous to an oligosaccharide derived from starch digestion, has an affinity for binding site of key carbohydrate hydrolysing enzymes. Such affinity is 10 000 to 100 000-fold higher than that of regular oligosaccharides from nutritional carbohydrates, and C–N linkage present cannot be cleaved, thus acting as a potent blocker of enzymatic hydrolysis [30]. These outcomes were in contrast to the study of Kotowaroo et al. [7], where increasing concentration of aqueous VM leaf extracts did not result in significant inhibitory action on the enzyme. Thus, it can be postulated that such significant inhibitory activity of the VM leaf decoction extract on *α*-amylase might be one reason that would validate its traditional use for diabetic management [1].

One could argue that various tested extracts of VM contain bioactive compounds that affect the activity of the two carbohydrate-hydrolyzing enzymes in several ways like competing with the substrate to bind with the active site of the enzyme or it might also work by binding to another region or to an enzyme substrate complex. Thus, kinetics parameters were calculated from the double reciprocal plot for the most active extracts. The trend lines revealed that both the maximal velocity of the enzyme-substrate reaction (*V*_max⁡_) and the affinity (*K*_*m*_) are decreased in the presence of the plant extracts, suggesting a mixed noncompetitive type of inhibition against both**α**-amylase and**α**-glucosidase. Mixed inhibition is a mode of enzyme inhibition whereby the inhibitor binds to the enzyme irrespective of whether the enzyme is already bound to the substrate or not, but it has a greater affinity for one state or the other. The noncompetitive inhibition exhibited by the extracts implies that they had different affinities for both the free enzymes and the enzyme-substrate complexes. Consequently, this suggests that the active component of the extract binds to a region other than the active site of the enzymes or combines with either free enzymes or enzyme-substrate complex possibly interfering with the action of both [31]. Therefore, this type of inhibition is said to result from an allosteric effect where the inhibitor binds to a different site on an enzyme, causing conformational changes that ultimately decrease affinity of the substrate to the active site. When the inhibitor favours binding to the enzyme-substrate complex, an increase in the 1/*K*_*m*_ value is noted which consequently suggest that affinity is reduced, causing decrease in velocity of the enzyme-substrate reaction [32]. In the same line of argument, the decrease in *K*_*m*_ and *V*_max⁡_ observed from the experimental data implied that the present kinetics study tends to suggest that the bioactive compounds in the extracts bind preferably to the enzyme-substrate complex. It is also worth highlighting that the plant inhibitors having mechanism of action of not occupying the active site or not competing with a substrate to bind to the active site of *α*-amylase offer major advantage over acarbose which is a competitive inhibitor. This also means that the action of the plant inhibitors would not be affected at higher concentration of substrate and would still be effective at lower concentration. In contrast, higher concentration of the acarbose would be needed to produce the same effect [33].

Published research suggests that there is a significant relationship between phenolic content, flavonoids, and other phytochemical compounds like condensed tannins in extract and the ability to inhibit *α*-amylase and *α*-glucosidase [34]. For instance, studies have found that flavonoids could demonstrate the highest inhibitory activities depending on the number of hydroxyl groups in the molecule of the compound. It was shown that the potency of inhibition is correlated with the number of hydroxyl groups on the B ring of the flavonoid skeleton [35]. In the present study, no correlation between neither total phenolic, flavonoid nor proanthocyanidins contents of the different extracts and the inhibition of *α*-glucosidase or pancreatic *α*-amylase was observed. Consequently, it can be assumed that active extracts may have other chemical components which play an important role in inhibition of *α*-glucosidase or *α*-amylase activities. None of the extracts, however, showed inhibitory effects in a dose response manner on increasing concentrations probably because of saturation at high concentrations thereby causing no further increase in inhibition [7].

Recently, many investigators have focused on the potential of different plant extracts on the diffusion of glucose across the semipermeable membrane or dialysis tube [36]. Such attention has probably been aroused by the fact that in recent years, national and international diabetes associations have consistently emphasized the need to increase intake of a high dietary fibre diet. The viscous and gel-forming properties of soluble dietary fibre like guar gum or *β*-glucan have been documented to be able to reduce macronutrient absorption, specifically postprandial glucose response after carbohydrate-rich meals and beneficially influence certain blood lipids [37]. In the present study, the* in vitro *dialysis-based model revealed that most of the different extracts did not significantly retard glucose movement across the dialysis tube. Though the exact mechanism of retardation was not investigated, it can be suggested that the concentration, pH, osmolarity, or water retention ability of soluble fibre present in the extract might act as important factors in the antihyperglycemic activity [38, 39]. It was also brought forward by Ahmed et al. [40] that the retardation in glucose diffusion might also be attributed to the physical obstacle presented by high molecular weight fiber particles towards glucose molecules and the entrapment of glucose within the network formed by fibers.

The present investigation has also endeavored to probe into the antimicrobial properties of VM using the disk diffusion assay and the determination of minimum inhibitory concentration. Results clearly demonstrate that out of the 8 extracts of VM investigated, only antimicrobial properties of the unripe decoction extract were active against the Gram negative* E. coli* whereas ripe decoction and seed methanol extracts were on the other hand active against Gram positive* S. aureus. *Leaf methanol extracts having the highest percentage activity were active against both bacteria. According to EUCAST [41], the antibiotic breakpoint assessed by the disc diffusion method was >26 mm for* S. aureus*, >14 mm for* E. coli*, and >15 mm for* P. aeruginosa* for detection of susceptible bacteria. Thus, results obtained concerning zone of inhibition of standard antibiotic used in this study fell within respective range and this confirmed susceptibility of these strains of bacteria. However, though certain extracts were active, zones of inhibition obtained were significantly less compared to the standard antibiotic used. None of the active extracts had antimicrobial potency comparable to the standard antibiotic. The plant extracts were less effective against the Gram negative bacteria probably because of their resistant multilayered structure of the Gram negative cell wall and their ability to form biofilms.* Per se*, it is documented that in the biofilms, the bacteria are embedded in an extracellular polymeric matrix and are protected against environmental stresses and antimicrobial treatment as well as against the host immune system [42]. Moreover, reports from the Centers for Disease Control and Prevention (CDC) and National Institutes of Health (NIH) estimated that the frequency of infections caused by biofilms, especially in the developed world, lies between 65% and 80%, respectively, [43]. Also, no activity was detected against fungi, which probably points out that antibacterial agents are more common in plants studied than antifungal agents [16].

Differences in antimicrobial property of the plant extracts probably related to the presence of bioactive compounds since an arsenal of phytochemicals originally serve as defense mechanisms against microbial predation [44]. Interestingly, phytochemical screening of the current investigation revealed that the active extracts possessed different amounts of at least 7 classes of bioactive metabolites: alkaloids, saponins, phenolic compounds, flavonoids, anthraquinones, anthocyanins, and to a lesser extent, steroids and saponins. For this case, toxicity of phenolic compounds to microorganisms is directly proportional to the degree of hydroxylation. The oxidised compounds can cause bacterial enzyme inhibition possibly through interaction with sulphydryl groups or bacterial proteins [44]. Quinones and flavonoids have the ability to complex irreversibly with nucleophilic amino acids in bacterial proteins like adhesions, cell wall envelope transport proteins, thereby causing their inactivation [44].

Studies have long established that ROS have potent oxidative effects on many cellular constituents (e.g., protein, lipids, and DNA), which leads to impairments of various cellular functions; thus they are directly and indirectly associated with the pathogenesis of insulin resistance via the inhibition of insulin signals and the dysregulation of adipocytokines/adipokines which have been implicated in the pathogenesis and progression of diabetes, hypertension, atherosclerosis, and cancer [45] or metabolic syndrome and the collection of cardiometabolic risk factors that include obesity, insulin resistance, hypertension, and dyslipidemia [46]. The scavenging of free radicals is thought to be a valuable measure to depress the level of oxidative stress in tissues for prevention and treatment of these chronic and degenerative diseases [47]. As a result, to further delineate any antioxidant effects of the 8 extracts of VM, 6 standard antioxidant assays were carried out.

Six standard antioxidant assays were performed to assess the various mechanisms of the antioxidant potential of VM. A strong significant correlation between FRAP and DPPH values suggested that antioxidant components in different VM extracts were capable of both reducing oxidants and scavenging free radicals. When considering the ferric reducing power, the methanol extracts had overall higher trolox equivalence which significantly differed from respective decoction extracts. Similar results were also noted with DPPH assay. The methanol extracts had significantly higher antioxidant capacity compared to decoction extracts which was probably due to the fact that methanol is more efficient in extracting polyphenols and anthocyanidins rather than a single-compound solvent system like water [48]. Methanol extracts had indeed the highest amount of total phenol content while the amounts of proanthocyanidins were also considerable. It is also worth noting that the presence of reducing sugars such as sucrose and fructose, ascorbic acid, aromatic amines, and some amino acids in extracts might also react with the Folin-Ciocalteu reagent, therefore leading to an overestimation of phenols [49]. Furthermore, to confirm whether the antioxidant potential of VM extracts is dependent on either total phenol, flavonoid, or proanthocyanidins contents, results revealed strong correlation between antioxidant capacities assessed by DPPH and FRAP and total phenolic content. It has been found that phenolics can scavenge DPPH radicals by their hydrogen donating ability [50].

Another important observation from the present study is the significant difference between the antioxidant property of unripe and ripe fruits when extracted with the same solvent. These results tend to corroborate with previous investigations, where they reported parallel results when comparing antioxidant activity of fruit extracts at different stages of ripening [50, 51]. Phenolic compounds are documented to synthesize rapidly during the early stages of fruit maturity. As the fruit matures, decline in phenolic compounds concentration is simultaneously observed due to the dilution caused by cell growth [50]. In fact, there is also a decrease of primary metabolism in the ripe fruit resulting in a lack of substrates essential for the biosynthesis of phenolic compounds. Besides transformation reactions like polymerisation, oxidation, and conjugation of bound phenolics during maturation could also result in the decrease of phenolic composition [51].

In the present study, methanol unripe fruit extract had the highest scavenging action but none comparable to ascorbic acid. Although no significant difference was obtained, flavonoids were shown to have a considerable percentage of shared variance with HOCl scavenging effect demonstrating moderate association. Indeed, according to Ribeiro et al. [52], flavonoids have the ability to modulate the neutrophil's oxidative burst. It was also demonstrated that flavonoids with either the catechol moiety or a* p*-unsaturated carbonyl with the free hydroxyl group at C-3 have shown the best myeloperoxidase inhibitory properties [53].

With regard to ^∙^OH, these are singlet oxygen species, which are highly reactive and have the capacity to damage DNA, which appears to represent the major target, involved in mutagenesis, carcinogenesis, diabetes, and so forth [23]. VM was found to remove the hydroxyl radicals from the sugar and prevented the reaction. The data proved that methanol extracts had better scavenging activity than decoction extracts with overall lower IC_50_ values. However, they were not a stronger scavenger of ^∙^OH compared to the *α*-tocopherol. Highest percentage of shared variance was obtained with total phenolic content which established that the scavenging effect was probably due to these bioactive compounds.

High concentration of nitric oxide produced by inducible nitric-oxide synthase in macrophages can result in oxidative damage through the conversion of peroxynitrite [54]. Sustained accumulation of this radical directly contributes to the vascular collapse associated with septic shock, whereas chronic expression of the NO radical is associated with a range of carcinomas and inflammatory conditions like juvenile diabetes, multiple sclerosis, arthritis, and ulcerative colitis [23]. The scavenging effect of plants extracts on NO was more pronounced in the methanol extracts. More specifically, methanol leaf extract had an IC_50_ value significantly lower than the control ascorbic acid, and thus, it might be suggested that it has a more potent NO scavenging activity than the standard.

With respect to iron chelating activity, only leaf decoction extracts had a scavenging effect significantly higher than the positive control EDTA. On the other hand, other extracts can be deemed to have comparable strong effects in stabilizing the oxidised form of the metal ion. Indeed, the two oxidation states of iron, Fe^2+^ and Fe^3+,^ can donate or accept electrons through redox reactions that are important for normal metabolic reactions, but in excess they also may be harmful to cells by aiding in the conversion of superoxide anion (O^*∙*2−^) and H_2_O_2_ to the extremely reactive ^∙^OH [55]. Such activity in this study has been mildly associated with flavonoids present in the extracts. As per Symonowicz and Kolanek [56]structural composition of several flavonoids revealed that there are three potential coordination sites to chelate metal ions, namely, between 5-hydroxy and 4-carbonyl groups, between 3-hydroxy and 4-carbonyl groups, and between 3′,4′-hydroxy groups in B ring.

## 5. Conclusions 

Though being an underutilized food plant, VM can be considered as a promising medicinal food plant that deserves to be further explored for the management of diabetes and related complications. Indeed, impeding the absorption of glucose through the inhibition of the carbohydrate-hydrolyzing enzymes such as *α*-amylase and *α*-glucosidase in the digestive tract could enable overall smooth glucose management in diabetic patients. VM extracts being more of the noncompetitive type inhibitor implies that the bioactive components responsible for such action would rather bind to a region beside the active site which is a major advantage over acarbose which is a competitive inhibitor. As a result, it is evident that, with higher intake of dietary carbohydrates, higher concentration of acarbose would be needed to show the same effect. This would not be the case with VM which is still effective at lower concentration. Given the dearth of updated information on the biological properties of VM, this study has provided an opportunity to establish valuable primary information on the bioactivity of VM and has opened new perspectives for further pharmacological research.

## Figures and Tables

**Table 1:** Inhibitory activity of VM extracts on *α*-amylase and *α*-glucosidase.

Extracts	IC_50_^a^ (mg/mL)	
	*α*-amylase	*α*-glucosidase
Decoction		
Leaf	1.12 ± 0.17^a^	0.61 ± 0.21^b^
Unripe fruit	5.25 ± 15.69^a^	0.50 ± 6.01^b^
Ripe fruit	29.62 ± 13.73^a^	15.73 ± 4.19^a^
Seed	6.81 ± 2.95^a^	182.14 ± 103.36^a^
Methanol		
Leaf	1.70 ± 0.10^a^	6.19 ± 1.87
Unripe fruit	1.23 ± 0.24^a^	0.36 ± 0.07^b^
Ripe fruit	7.74 ± 1.56^a^	3.28 ± 0.45^b^
Seed	3.75 ± 1.18^a^	46.28 ± 6.01^a^
Acarbose	0.11 ± 0.03	5.03 ± 0.14

^a^IC_50_ is defined as the concentration sufficient to obtain 50% of maximum inhibitory activity, expressed as mean ± SD (*n* = 3). ^a^ *P* < 0.05 is considered as significantly higher from positive control acarbose (400 *μ*g/mL). ^b^ *P* < 0.05 is considered as significantly lower from positive control acarbose.

**Table 2:** Relationship between phytochemical constituents and key carbohydrate enzymes inhibitory effects of extracts.

Phytochemical constituent	*α*-amylase IC_50_^a^ (µg /mL)		*α*-glucosidase IC_50_^a^ (µg /mL)	
	*r *	Sig. (2-tailed) value	*r *	Sig. (2-tailed) value
Total phenolic content ^1^	−0.20	>0.05	0.98	>0.05
Total flavonoid^2^	−0.99	>0.05	0.57	>0.05
Total proanthocyanidins^3^	−0.56	>0.05	0.99	>0.05

^a^IC_50_ is defined as the concentration sufficient to obtain 50% of maximum scavenging activity, expressed as mean ± SD (*n* = 3). *r* = Pearson correlation.^ 1^mg GAE/g fresh weight; ^2^mg RE/g fresh weight; ^3^mg CE/g fresh weight.

**Table 3:** Effect of VM on the movement of glucose over 3 hrs incubation.

Extracts	Concentration of glucose in external solution (mM/L) after 1 hr incubation period						
	0	0.5	1	1.5	2	2.5	3
Decoction							
Leaf	2.17 ± 0.087	2.89 ± 0.15^b^	3.68 ± 0.07^b^	4.63 ± 0.093^ab^	4.22 ± 0.097*	4.04 ± 0.035^∗a^	3.76 ± 0.080^∗a^
Unripe fruit	2.18 ± 0.061	3.06 ± 0.22	3.73 ± 0.08	3.79 ± 1.89	4.39 ± 0.16	4.20 ± 0.046^∗b^	4.54 ± 0.046^∗b^
Ripe fruit	2.23 ± 0.046	3.42 ± 0.14	3.87 ± 0.05	4.31 ± 0.31	5.53 ± 0.076	5.96 ± 0.063	8.37 ± 0.61^b^
Seed	2.25 ± 0.035	3.51 ± 0.076^b^	3.99 ± 0.046	4.68 ± 0.063	5.09 ± 0.061	5.67 ± 0.08	6.49 ± 0.122
Methanol							
Leaf	2.22 ± 0.087	2.72 ± 0.25	3.37 ± 0.093*	3.75 ± 0.063^∗ab^	4.13 ± 0.046*	4.70 ± 0.380^∗ab^	5.04 ± 0.046^∗a^
Unripe fruit	2.33 ± 0.052	2.79 ± 0.061	4.37 ± 0.12^b^	4.47 ± 0.076	4.91 ± 0.061^b^	5.68 ± 0.046	8.02 ± 0.23^b^
Ripe fruit	2.31 ± 0.076	3.10 ± 0.076	3.54 ± 0.19	4.39 ± 0.061	5.88 ± 0.24^b^	6.54 ± 0.11	8.42 ± 0.24^∗b^
Seed	2.28 ± 0.017	2.81 ± 0.061	3.55 ± 0.061	4.34 ± 0.076	4.93 ± 0.178	5.41 ± 0.070	7.30 ± 0.33^b^
Blank	2.26 ± 0.017	2.85 ± 0.03^b^	4.03 ± 0.091^b^	4.48 ± 0.080^b^	5.30 ± 0.052^b^	6.01 ± 0.24^b^	7.16 ± 0.16^b^

All data are shown as mean ± SD; each run in triplicates; *P < 0.05 is considered as statistically significant (one way ANOVA with post hoc analysis) compared to blank/negative control at respective time interval. ^a^Significant difference (*P* < 0.05) exists between leaf decoction and leaf methanol extracts at respective time interval. ^b^ *P >* 0.05 compared with glucose concentration at a previous time of incubation.

**Table 4:** Results of preliminary antimicrobial screening of the plant extracts (50 mg/mL) using disc diffusion method.

Test microorganisms	Gram stain +/−	Standard^b^	Diameter of zone of inhibition (mm)^a^							
			Decoction^c^				Methanol^c^			
			S1	S2	S3	S4	S1	S2	S3	S4
*Staphylococcus aureus *	G+	26.33 ± 0.58	—	—	10.67 ± 1.15^d^	—	11.67 ± 1.53^d^	—	—	8.33 ± 1.53^d^
*Escherichia coli *	G−	21.67 ± 3.79	—	12.67 ± 0.58^d^	—	—	10.00 ± 2.00^d^	—	—	—
*Pseudomonas aeruginosa *	G−	15.33 ± 1.53	—	—	—	—	—	—	—	—
*Aspergillus niger *	F	23.00 ± 1.00	—	—	—	—	—	—	—	—
*Candida albicans *	F	20.67 ± 0.58	—	—	—	—	—	—	—	—

^a^No. of replicates (*n* = 3) for each sample; values are given as mean ± SD. ^b^Tested at a concentration of 10 µg/disk (Oxoid), bacteria, ampicillin; fungi, nystatin. ^c^S1: leaf, S2: unripe fruit, S3: ripe fruit, S4: seed. ^d^Values significantly lower (*P* < 0.05) from positive control, standard antibiotic (One way ANOVA, post hoc Tukey). G+, Gram positive; G−, Gram negative; F, fungi; (−), no distinct zone of inhibition.

**Table 5:** Minimum inhibitory concentrations (mg/mL) of the plant extracts.

Test microorganisms	Gram stain +/−	Standard antibiotic^b^ (mg/mL)	Plant extracts^c^ [MIC^a^ (mg/mL)]							
			Decoction				Methanol			
			S1	S2	S3	S4	S1	S2	S3	S4
*Staphylococcus aureus *	G+	0.078	—	—	12.50	—	6.25	—	—	25.00
*Escherichia coli *	G−	0.078	—	25.00	—	—	12.50	—	—	—

^a^MIC: minimum inhibitory concentration; average of 3 independent experiments; ^b^Streptomycin sulphate and gentamicin sulphate tested at a concentration of 20 mg/mL; ^c^S1: leaf, S2: unripe fruit S3: ripe fruit, S4: seed; G+, Gram positive; G−, Gram negative.

**Table 6:** DPPH scavenging activity of plant extracts.

Samples	IC_50_^a^ (µg/mL)		*F *	One way ANOVA
	Decoction	Methanol		*P* value (post hoc)
Leaf	132.78 ± 11.38^a^	9.04 ± 0.66	349.97	<0.05*
Unripe fruit	612.46 ± 47.21^a^	10.01 ± 0.93		<0.05*
Ripe fruit	602.54 ± 39.53^a^	48.46 ± 0.63		<0.05*
Seed	612.46 ± 47.22^a^	105.86 ± 2.82^a^		<0.05*

^a^IC_50_ is defined as the concentration sufficient to obtain 50% of maximum scavenging activity, expressed as mean ± SD (*n* = 3). *P < 0.05 is considered as statistically significant (post hoc Tukey HSD). ^a^Values significantly higher (*P* < 0.05) from positive control, ascorbic acid (IC_50_ = 0.001 ± 0.0006 µg/mL).

**Table 7:** Ferric reducing antioxidant power of extracts.

Samples	mM trolox equivalent (TE)/g fresh weight^a^		*F *	One way ANOVA
	Decoction	Methanol		*P* value (post hoc)
Leaf	350.42 ± 1.91	372.5 ± 2.17	186.81	<0.05*
Unripe fruit	330.83 ± 2.83^b^	361.25 ± 1.25		<0.05*
Ripe fruit	322.93 ± 0.72^b^	357.08 ± 0.72		<0.05*
Seed	319.17 ± 5.05	346.67 ± 1.91		<0.05*

^a^Data are expressed as mM trolox equivalent (TE)/g fresh weight, mean ± SD (*n* = 3). ^b^Significant difference (*P* < 0.05) exists between unripe fruit and ripe fruit extracts within same extraction solvent. *P < 0.05 is considered as statistically significant (post hoc Tukey HSD).

**Table 8:** Relationship between phenolic content and antioxidant activity of extracts.

Phytochemical constituent	Pearson correlation			
	DPPH^a^IC_50_^b^ (µg/mL)		FRAP^a^ mM trolox equivalent (TE)/g fresh weight	
	*r *	Sig. (2-tailed) value	*r *	Sig. (2-tailed) value
Total phenolic content (mg GAE/g fresh weight)	−0.78	<0.05*	0.88	<0.05*
Total flavonoid (mg RE/g fresh weight)	−0.28	>0.05	0.49	>0.05
Total proanthocyanidins (mg CE/g fresh weight)	−0.40	>0.05	0.54	>0.05

^b^IC_50_ is defined as the concentration sufficient to obtain 50% of maximum scavenging activity, expressed as mean ± SD (*n* = 3). *P < 0.05 is considered as statistically significant. ^a^Correlation coefficient of DPPH-FRAP: *r* = −0.94, *P* < 0.05.

**Table 9:** Scavenging of reactive oxygen species and iron chelating activity (IC_50 _values) of extracts and reference compounds.

Activity	Extract	IC_50_^a^ (µg /mL)		One way ANOVA	
		Decoction extracts	Methanolic extracts	*F *	*P* value (post hoc)
HOCl	Leaf	235.55 ± 10.61	382.06 ± 4.35	682.92	>0.05
	Unripe fruit	275.27 ± 18.21^c^	222.99 ± 3.15		>0.05
	Ripe fruit	982.44 ± 70.66^bc^	418. 91 ± 39.22		<0.05*
	Seed	6656.35 ± 390.40^b^	941.50 ± 120.40^b^		<0.05*

^∙^OH	Leaf	289.04 ± 5.29^d^	0.09 ± 0.04	3.03	<0.05*
	Unripe fruit	157.21 ± 1.19^cd^	0.29 ± 0.08		<0.05*
	Ripe fruit	260.96 ± 4.29^cd^	0.26 ± 0.02		<0.05*
	Seed	803.76 ± 23.72^d^	22.43 ± 3.97		<0.05*

NO	Leaf	241.22 ± 34.74	43.22 ± 0.59^f^	434.23	>0.05
	Unripe fruit	436.24 ± 2.99^c^	91.36 ± 3.26		>0.05
	Ripe fruit	2367.36 ± 198.63^ce^	219.14 ± 39.78		<0.05*
	Seed	6092.38 ± 443.32^e^	1103.20 ± 11.80^e^		<0.05*

Iron chelating^g^	Leaf	2.52 ± 1.76^h^	0.002 ± 0.0005	4.96	<0.05*
	Unripe fruit	0.95 ± 0.40	0.07 ± 0.03		>0.05
	Ripe fruit	0.57 ± 0.52	0.06 ± 0.04		>0.05
	Seed	0.25 ± 0.42	0.0009 ± 0.0003		>0.05

^a^IC_50_ expressed as mean ± SD (*n* = 3). ^b^Values significantly higher (*P* < 0.05) from ascorbic acid (400 µg/mL; IC_50_ = 46.00 ± 2.35 µg/mL). ^c^Significant difference (*P* < 0.05) exists between unripe fruit and ripe fruit extracts within same extraction solvent. ^d^Values significantly higher (*P* < 0.05) from *α*-tocopherol (400 µg/mL; IC_50_ = 0.50 ± 0.11 µg/mL). ^e^Values significantly higher (*P* < 0.05) from ascorbic acid (400 µg/mL; IC_50_ = 546.54 ± 9.79 µg/mL). ^f^Value significantly lower (*P* < 0.05) from ascorbic acid (400 µg/mL; IC_50_ = 546.54 ± 9.79 µg/mL). ^g^IC_50_ values expressed in mg/mL. ^h^Values significantly higher (*P* < 0.05) from EDTA (400 µg/mL; IC_50_ = 0.001 ± 0.0003 µg/mL). *P < 0.05 is considered as statistically significant (post hoc Tukey HSD).

**Table 10:** Total phenolic, flavonoid, and proanthoyanidin contents of extracts.

Plant extracts	Decoction	Methanol	*F *	One way ANOVA
				*P* value (post hoc)
	Total phenolic content (mg GAE/g fresh weight)^a^			
Leaf	58.56 ± 1.17	122.22 ± 1.02	1.16	<0.05*
Unripe fruit	35.00 ± 0.33^d^	70.33 ± 0.33^d^		<0.05*
Ripe fruit	37.00 ± 0.88^d^	61.22 ± 1.07^d^		<0.05*
Seed	35.67 ± 0.33	67.33 ± 3.53		<0.05*

	Total flavonoid content (mg RE/g fresh weight)^b^			
Leaf	8.90 ± 0.35	9.00 ± 0.05	61.06	>0.05
Unripe fruit	8.43 ± 0.18	7.55 ± 0.26^d^		<0.05*
Ripe fruit	8.00 ± 0.13	8.20 ± 0.07^d^		>0.05
Seed	6.72 ± 0.04	7.13 ± 0.13		>0.05

	Total proanthocyanidins (mg CE/g fresh weight)^c^			
Leaf	159.32 ± 5.43	185.72 ± 1.14	563.37	<0.05*
Unripe fruit	78.65 ± 2.86^d^	154.92 ± 3.54^d^		<0.05*
Ripe fruit	159.50 ± 2.75^d^	134.57 ± 2.60^d^		<0.05*
Seed	60.87 ± 4.41	42.53 ± 6.06		<0.05*

All data are shown as mean ± SD in triplicates; ^a^data are expressed as mg gallic acid equivalent (GAE)/g fresh weight; ^b^data are expressed as mg rutin equivalent (RE)/g fresh weight; ^c^data are expressed as mg catechin equivalent (CE)/g fresh weight; ^ d^significant difference (*P* < 0.05) exists between ripe fruit and unripe fruit samples extracted using same solvent. Refer to text. *P < 0.05 is considered as statistically significant.

**Table 11:** Qualitative phytochemical screening of the plant extracts.

Bioactive compounds	Plant extracts^a^							
	Decoction				Methanolic			
	S1	S2	S3	S4	S1	S2	S3	S4
Alkaloids	−	+	+	−	++	+	+	−
Saponins	−	+	−	+	−	−	−	−
Phenolic compounds	+++	++	++	++	+++	+++	++	++
Flavonoids	+	+	+	+	++	+	+	+
Anthraquinones	+	+	−	−	+	+	+	−
Steroids	−	−	−	+	+	−	−	+
Anthocyanins	++	+	++	+	++	++	++	+

(−): Absence, (+): low presence, (++): moderate presence, (+++): high presence. ^a^S1: leaf sample, S2: unripe fruit sample, S3: ripe fruit sample, S4: seed sample.
